# Supplementary material for: Complexome profiling of the Chlamydomonas psb28 mutant reveals TEF5 as an early PSII assembly factor
Source: Plant Cell. 2025 Mar 17;37(6):koaf055. doi: 10.1093/plcell/koaf055 (PMC12164590; doi:10.1093/plcell/koaf055)
Supplement: koaf055_Supplementary_Data [file koaf055_supplementary_data.zip › psb28_interactive.zip!/plots/intensity/qualityPlots/peptides/LHCA2_Cre12.g508750.t1.2.html]

### Cre12.g508750.t1.2

Trivial name: LHCA2  
SeqCoverage [median]: 49.60  
Mass: 26.92 kDa  
  
 

| Sequence | MissedCleavage | Mass [Da] | wtIntensity | muIntensity | Score |
| --- | --- | --- | --- | --- | --- |
| EAELTNGR | 0 | 888.43 | 3516.67 | 19071.62 | 150.84 |
| EAELTNGRWAMAAVVGILFTDAVGLPK | 1 | 2828.49 | 2020.62 | 0.00 | 64.68 |
| ESVPYFPWNEPWNKV | 1 | 1890.89 | 53240.92 | 0.00 | 160.91 |
| FWTAGAEK | 0 | 908.44 | 354837.25 | 129834.23 | 145.34 |
| KTGETGFLSFAPFDPMGMK | 1 | 2059.97 | 34052.77 | 0.00 | 101.17 |
| KTGETGFLSFAPFDPMGMKSEEMK | 2 | 2664.23 | 25799.71 | 27557.56 | 75.18 |
| LGVNKDNLK | 1 | 999.57 | 612456.55 | 459287.91 | 235.15 |
| RYEIYK | 1 | 870.46 | 86548.89 | 47562.14 | 143.02 |
| SEEMKLK | 1 | 863.44 | 401.28 | 18499.30 | 127.84 |
| SEEMKLKELK | 2 | 1233.66 | 616.69 | 7133.90 | 71.35 |
| TGETGFLSFAPFDPMGMK | 0 | 1931.88 | 204844.31 | 142668.21 | 227.48 |
| TGETGFLSFAPFDPMGMKSEEMK | 1 | 2536.13 | 137801.01 | 12976.36 | 149.36 |
| TGETGFLSFAPFDPMGMKSEEMKLK | 2 | 2777.31 | 77906.81 | 72342.32 | 102.16 |
| TLNPGKESVPYFPWNEPWNK | 1 | 2402.17 | 1215.95 | 0.00 | 49.77 |
| TLNPGKESVPYFPWNEPWNKV | 2 | 2501.24 | 30131.94 | 0.00 | 125.23 |
| WAMAAVVGILFTDAVGLPK | 0 | 1958.07 | 13638.16 | 0.00 | 148.03 |
| YALDNQTLALIEVAVFAVLEGKR | 1 | 2532.40 | 2724.76 | 0.00 | 199.60 |
| YEIYKK | 1 | 842.45 | 2613.84 | 44960.95 | 117.70 |
|  |  |  | 1644368.1 | 981894.5 |  |
